# A toxic burden in Ningbo, Zhejiang Province: elevated heavy metal concentrations in sanitation workers

**DOI:** 10.3389/fpubh.2026.1728011

**Published:** 2026-02-03

**Authors:** Zhen Li, Li Wang, Keye Xu, Yanyan Lu, Dandan Zhang

**Affiliations:** 1 Baihe Street Community Health Services of Yinzhou District, Ningbo, Zhejiang, China; 2 Department of Physical and Chemical Examination, Ningbo Municipal Center for Disease Control and Prevention, Ningbo, Zhejiang, China; 3 Department of Microbiology, Ningbo Municipal Center for Disease Control and Prevention, Ningbo, Zhejiang, China

**Keywords:** heavy metals, sanitation workers, serum, urine, whole blood

## Abstract

**Background:**

Sanitation workers are exposed to heavy metals due to improper disposal of waste. This study analyzes heavy metal concentrations in the serum, urine, and blood of sanitation workers exposed to improperly disposed e-waste and industrial residues, and examines how protective measures affect these levels.

**Methods:**

This study examines heavy metal levels in 102 sanitation workers (22 operators, 37 drivers, and 43 sanitary workers) versus a control group of 50 unrelated workers. Samples were pre-treated with 0.5% nitric acid and Triton X-100 for serum and blood, and 0.5% nitric acid with 200 μg/L gold for urine. ICP-MS in KED mode was used to measure concentrations of 10 heavy metals (Cr, Mn, Co, Ni, As, Cd, Sn, Sb, Hg, and Pb) in the samples.

**Results:**

Long working hours, working at disposal and hand injury were associated with higher concentrations of Cd, Sb, and Hg in human blood. Elevated serum levels of Mn (2.46 μg/L), Cd (0.14 μg/L), Sn (1.19 μg/L), and Sb (9.99 μg/L) were observed in sanitation workers. Among drivers, increased serum concentrations were noted for Mn (2.67 μg/L), Cd (0.15 μg/L), Sn (1.18 μg/L), and Hg (0.57 μg/L). Serum Sb (10.10 μg/L) was particularly elevated in the sanitary workers. Sanitation workers exhibited heightened urine levels of Cr (1.91 μg/L), Co (0.35 μg/L), Ni (2.79 μg/L), Cd (0.92 μg/L), Sn (6.43 μg/L), and Sb (0.14 μg/L). Operators demonstrated an increased concentration of Cr (2.37 μg/L). Drivers showed elevated levels of As (95.29 μg/L), Cd (1.34 μg/L), Sb (0.14 μg/L), and Hg (0.31 μg/L). Sanitary workers exhibited higher concentrations of Co (0.41 μg/L), Ni (3.54 μg/L), and Sn (6.79 μg/L). Blood concentration levels of As (8.70 μg/L), Cd (3.62 μg/L), Hg (3.82 μg/L), and Pb (16.71 μg/L) were highest in drivers’ group followed by operators, while lowest in sanitary workers. The median concentrations were all below the BEI (ACGIH, GBZ, WS/T).

**Conclusion:**

Sanitation workers face an elevated risk of exposure to heavy metals, may posing significant threats to their occupational health. There is a critical need for comprehensive prevention and intervention strategies to mitigate heavy metal exposure among sanitation workers.

## 1 Introduction

In recent years, human biomonitoring (HBM) has gained prominence as a method for assessing the health impacts of chemical exposure within the general population. Blood and urine are the primary matrices utilized in HBM to quantify chemicals and their metabolites in biological specimens (1, 2). Heavy metals, defined as metals with a density exceeding 5 g/cm^3^ (3), have emerged as a significant area of concern due to their origins in both natural processes and anthropogenic activities (4). Heavy metal pollution encompasses elements such as mercury (Hg), lead (Pb), cadmium (Cd), chromium (Cr), and arsenic (As), which are non-essential and highly toxic to humans (5). These non-degradable contaminants accumulate progressively in the body, depositing in adipose tissue, muscles, bones, and joints (6). Exposure to heavy metals can occur through various sources such as air, food, drinking water, smoking, cosmetics, dental amalgam, and other household products (7). The accumulation of heavy metals in bodily organs can result from prolonged exposure, presenting significant health challenges (8–10). Cd accumulation can detrimentally affect renal, skeletal systems and lung cancer (11); Cr exposure is associated with adverse respiratory outcomes (12); and nickel (Ni) exposure is a primary cause of contact allergies (13). The toxicity of Pb arises by disrupting physiological systems such as the nervous, cardiovascular, and hematopoietic systems, resulting in neurological damage, hypertension, and anemia, alongside reproductive effects like miscarriage and premature birth (10, 14, 15). The excessive accumulation of thallium in the body can lead to a range of disorders, immune systems, mental disorders, potential DNA damage, and cancer (16, 17).

**Figure 1:**
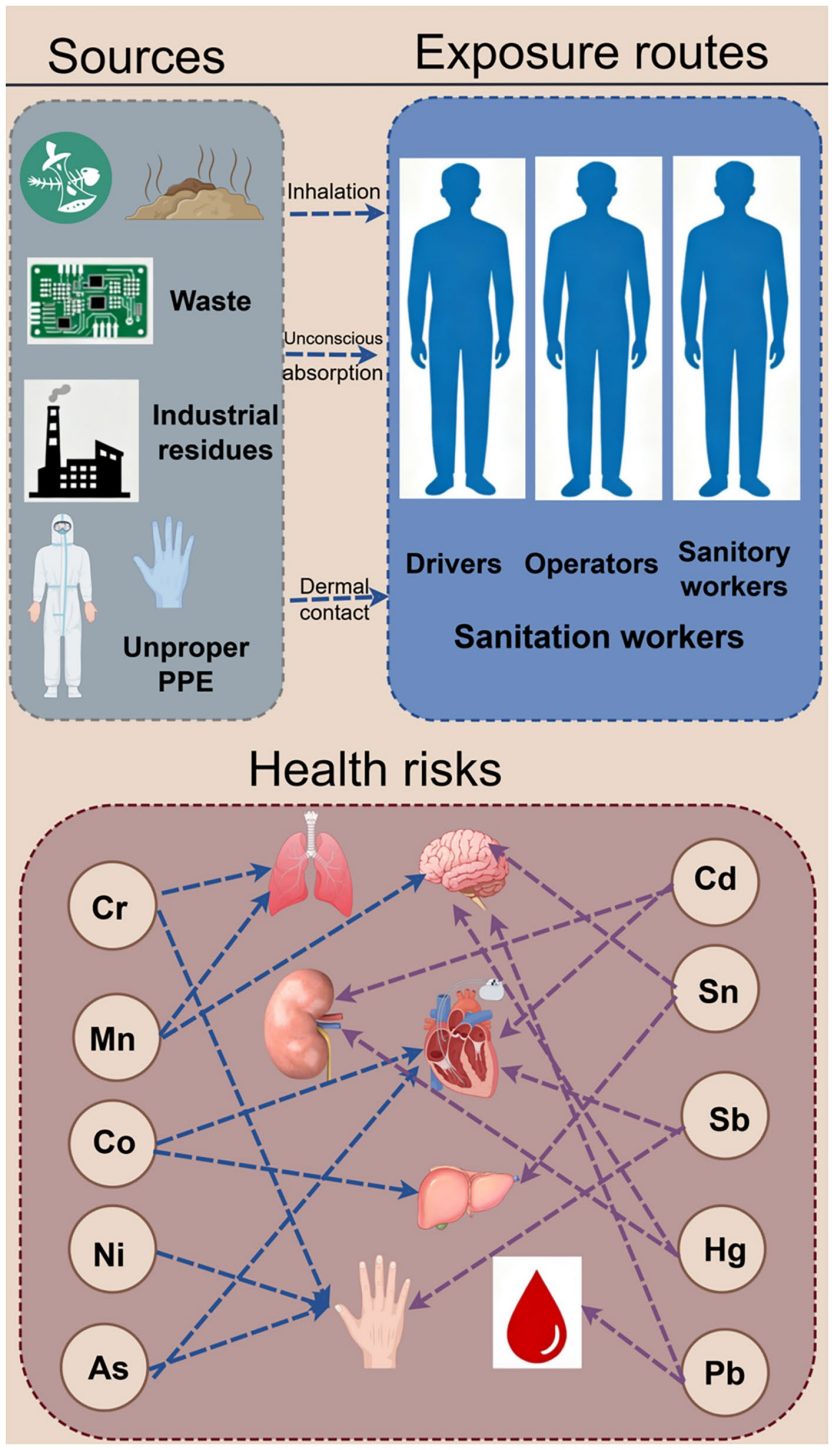
Schematic overview of heavy metal exposure and health risks for sanitation workers.

Sanitation workers encounter heavy metals through diverse exposure pathways (Figure 1). The improper disposal of waste materials, including electronic waste (e-waste) and industrial residues, constitutes a primary source of contamination. In India, informal e-waste recycling has become a notable contributor to environmental pollution, with heavy metals posing substantial health risks to workers involved in waste management (18). These workers may be exposed to heavy metals via inhalation, dermal contact, and ingestion during waste management activities such as incineration, landfilling, and sorting. Additionally, heavy metals present in soil and leachate at landfill sites can enter the body through dermal absorption or accidental ingestion. In an investigation conducted at an e-waste recycling site in Agbogbloshie, Ghana, biological samples including blood, urine, and hair from 75 e-waste workers were analyzed and compared. The median blood lead level was 88.5 μg/L among e-waste workers, in contrast to 41.0 μg/L in the control group, suggesting a heightened risk of heavy metal exposure for these workers (19). Furthermore, a study involving 254 sanitation workers in Guangzhou, China, assessed 10 metals in urine and evaluated the associated health effects. Abnormalities in maximal mid-expiratory flow (MMEF75/25) and maximal expiratory flow at 25% of vital capacity (MEF25) have been correlated with exposure to specific heavy metals, indicating that such exposure may detrimentally impact the health of sanitation workers (20). The pathways through which various heavy metals enter the human body differ, and once inside, these metals undergo intricate metabolic processes, exerting toxicological effects. Cd predominantly accumulates in the liver and kidneys, where it binds to metallothionein, disrupts intracellular redox homeostasis, and induces oxidative stress damage (21). Moreover, heavy metals may have prolonged effects on human health by modulating cellular signaling pathways, gene expression, and other molecular mechanisms. Certain heavy metals can activate the mitogen-activated protein kinase (MAPK) signaling pathway, inducing inflammatory responses and apoptosis, or they may modify gene expression through epigenetic changes, thereby elevating disease risk. This study aims to conduct an analysis among sanitation workers to investigate the concentrations of various heavy metals in their serum and urine, as well as to examine the relationship between protective measures and biological levels.

## 2 Methods

### 2.1 Reagents and materials

The mixed standard solutions comprising nine heavy metals—Cr, Mn, Co, Ni, As, Cd, Sn, antimony (Sb), and Pb were obtained from the National Center of Analysis and Testing for Nonferrous Metals and Electronic Materials (Beijing, China), in accordance with the GSB04-1767-2004 specification, each at a concentration of 100 μg/mL. Additionally, Hg and gold (Au) were procured at concentrations of 1,000 μg/mL, following the GSB04-1729-2004 and GSB04-1715-2004 standards, respectively. The mixed internal standard solution, containing scandium (Sc), germanium (Ge), indium (In), rhodium (Rh), and rhenium (Re), was also acquired, each at a concentration of 1,000 μg/mL, as per the respective GSB04 standards. Ultrapure water was sourced from a Milli-Q EQ7008 system (Merck, Germany). Suprapur^®^ nitric acid (68%, upgrade) was purchased from Jingrui Company (Jiangsu, China), while Triton X-100 (analytical reagent) was obtained from Macklin Company (Shanghai, China). A multi-element standard curve, including 10 elements—Cr, Mn, Co, Ni, As, Cd, Sn, Sb, and Pb—was prepared by diluting the standards with 1% upgraded nitric acid. A portion of the mixture was continuously diluted with 0.5% nitric acid to achieve final concentrations of 0, 0.1, 0.2, 0.5, 1.0, 10.0, 25.0, and 50.0 μg/L. A standard curve for Hg was prepared by diluting with 0.5% nitric acid, along with 200 μg/mL of Au. The points on the standard curve were set at 0.05, 0.1, 0.2, 0.5, 1, and 2 μg/L. Sc, Ge, In, Rh, and Re were selected as internal standard elements which were diluted with 0.5% nitric acid to a final concentration of 10 μg/L.

### 2.2 Determination of urinary and serum heavy metals

A total of 102 sanitation workers (22 operators, 37 drivers and 43 sanitary workers) and 50 non-related workers (control group) were included based on physical examination. The heavy metal concentration in serum was measured following the standard protocols of our laboratory. Briefly, all serum samples were collected at the hospital in single-use containers. The samples were transferred to the laboratory in less than 2 h and kept at 4 °C until analysis. 0.25 mL serum samples or whole blood samples were added into a plastic container with 4.75 mL 0.5% nitric acid and 0.01% Triton X-100 (Macklin, Shanghai, China), followed by vortexing for analysis. Urine samples were collected and transferred into 10-mL plastic vials with lids, subsequently stored at −20 °C until analysis. In brief, 0.5 mL of urine was combined with 4.5 mL of a solution containing 0.5% nitric acid and 200 μg/L gold, and the mixture was vortexed prior to analysis. Coupled Plasma Mass Spectrometry (ICP-MAS) (iCAP TQ, Thermofisher, United States) was used. KED mode was set to detect the concentration of different heavy metal in human serum and urine samples. The blank solution was double diluted water (DDW) and was prepared in the same manner as the experimental samples. Blank, multiple-elements standard, and biosamples were then subjected to analysis. All laboratory equipment and containers were washed with dilute HNO_3_ and rinsed with ultrapure water (18.2 MΩ cm) prior to use.

Good linearity was obtained from the calibration curves prepared from metals standard. The method detection limits (MDLs) were 0.8 μg/L for Cr, 0.14 μg/L for Mn, 0.024 μg/L for Co, 0.21 μg/L for Ni, 0.10 μg/L for As, 0.065 μg/L for Cd, 0.18 μg/L for Sn, 0.028 μg/L for Sb, 0.031 μg/L for Hg, and 0.047 μg/L for Pb.

### 2.3 Study population and sampling

This study was conducted on sanitation workers from July to September 2024, following their annual physical examinations. Samples were collected from the urine, serum and whole blood of 102 sanitation workers, comprising 91 males (89.22%) and 11 females (10.78%), at the Environmental Sanitary Management Center, which oversees waste disposal in the Haishu district, located in the western part of Ningbo City, Zhejiang Province. The study participants categorized as “sanitation workers” were recruited from the municipal solid waste management system. This cohort comprised three distinct occupational subgroups based on their specific job duties and exposure environments: Street Sanitary Workers: individuals responsible for manual sweeping and collection of refuse from public streets and sidewalks. Drivers: Personnel operating collection vehicles to transport municipal solid waste from residential and commercial areas to centralized sorting stations. Operators: Workers stationed at waste sorting facilities, tasked with the manual sorting and handling of mixed municipal solid waste upon its arrival. The study involved two groups: the case group consisted of individuals involved in environmental cleaning, including operators, drivers, and sanitation workers, while the control group was randomly selected from a physical examination center and comprised individuals not engaged in sanitation work. In total, our study included 102 sanitation workers and 50 participants in the control group. Consequently, we collected 102 serum samples, 102 urine samples, and 102 whole blood samples from the sanitation workers, and 50 serum samples and 38 urine samples from the control group. At the commencement of the study, participants completed a questionnaire that included informed consent and demographic information, such as age and occupation.

### 2.4 Inclusion and exclusion criteria

The inclusion criteria for the study were defined as follows: (1) a minimum of 1 year of experience in environmental cleaning or a related field, (2) completion of the informed consent process, and (3) an age range of 17–70 years. The exclusion criteria included: (1) a self-reported history of heavy metal poisoning, (2) occupational exposure to heavy metals in industries such as battery manufacturing, soldering, painting, crystal production, cement factories, steel factories, and power plants, (3) current drug use, and (4) a history of psychiatric disorders or mental health issues.

### 2.5 Statistical analysis

All figures and data were generated using R software (Version 4.2.1).^1^ Descriptive statistics were analyzed using the Kruskal-Wallis rank sum test (non-normally distributed data) with statistical significance determined at a *p*-value of less than 0.05 or an adjusted *p*-value of less than 0.05. A Bonferroni correction was applied to adjust *p*-values for the three primary pairwise comparisons following a significant Kruskal-Wallis test while the Wilcoxon test was used for group comparisons (Cr, Cd, Hg, and Pb in serum group; Cr, Mn, Co, Ni, Cd, Sb, and Hg in urine group) involving data below the method detection limit (MDL). For multivariable regression analysis of metal concentrations below the limit of detection (LOD), Tobit regression was utilized to account for left-censored data. This method avoids the bias associated with simple value substitution and provides consistent estimates of the associations between exposures and outcomes. To contextualize the measured concentrations, the findings were compared against the biological reference values established by the American Conference of Governmental Industrial Hygienists (ACGIH), national standards GBZ 2.1–2019 and Chinese national recommended standards (WS/T).

## 3 Results

### 3.1 Characteristics of study participants

Table 1 provides a comprehensive statistical summary of the descriptive characteristics of the study participants, categorized into distinct groups. The study included a total of 102 sanitation workers, consisting of 22 operators, 37 drivers, and 43 sanitary workers, in addition to a control group of 50 unrelated individuals. Within the sanitation worker cohort, there was one female operator and 11 female sanitary workers. The control group was randomly selected from a physical examination center, and all individuals in this group remained anonymous, precluding the collection of personal information such as age and sex. The mean ages for the sanitation workers, operators, drivers, and sanitary workers were 52.51 ± 5.50, 53.23 ± 7.02, 52.14 ± 4.57, and 52.40 ± 5.16 years, respectively, as detailed in Table 1.

**Table 1:** Descriptive statistics of 102 participants included in study.

Character	Operators	Drivers	Sanitary workers	Sanitation workers	Control group
Age	53.23 ± 7.02	52.14 ± 4.57	52.40 ± 5.16	52.51 ± 5.50	/
Male (*n*^1^)	21	37	33	91	/
Female (*n*^2^)	1	0	11	11	/
Total (N)	22	37	43	102	50

Data are presented as mean ± standard deviation (SD). n^1^ denotes the number of males in each group, and n^2^ denotes the number of females in each group. A total of 102 sanitation workers included 22 operators, 37 drivers and 43 sanitary workers. The control group were anonymous which were collected from a routine health screening biobank.

### 3.2 Multivariable regression analysis of risk factors

The questionnaire items are comprehensively detailed in our previous publication (22). The most commonly reported risk factors were extended working hours (60.78% affirmative responses) and employment in waste management (52.94% affirmative responses). The findings from the multivariable linear regression analysis are illustrated in Supplementary Figures 1–3. Three factors emerged as statistically significant independent predictors. A history of hand injury (Hg, *β* = 0.154, *p* = 0.017) (Pb, *β* = 0.091, *p* = 0.046), the use of boots during work (Mn, *β* = 0.355, *p* = 0.042) were both associated with elevated concentrations of Hg, Pb and Mn in human serum. Extended working hours were linked to a reduction in Co concentration in human serum (*β* = −0.083, *p* = 0.046). Interestingly, a history of hand wounds (Cr, *β* = −0.707, *p* = 0.026) (Sn, *β* = −1.375, *p* = 0.016), foot wounds (Pb, β = −0.465, *p* = 0.047), and employment in waste management (Sn, *β* = −1.122, *p* = 0.049) were all correlated with a significant decrease in Cr, Sn and Pb levels in human urine. Conversely, the use of gloves during work was associated with an increase in Sb levels in human urine (*β* = 0.249, *p* = 0.041). The use of mask (Cd, *β* = 1.111, *p* = 0.037), long working hours (Sb, *β* = 0.896, *p* = 0.003), working at garbage disposal (Sb, *β* = 0.691, *p* = 0.019), hand wounds (Hg, *β* = 0.912, *p* = 0.039) were associated with increased concentrations of Cd, Sb, Hg in human whole blood.

### 3.3 Concentration levels of heavy metals in human serum between sanitation workers and control group

**Figure 2:**
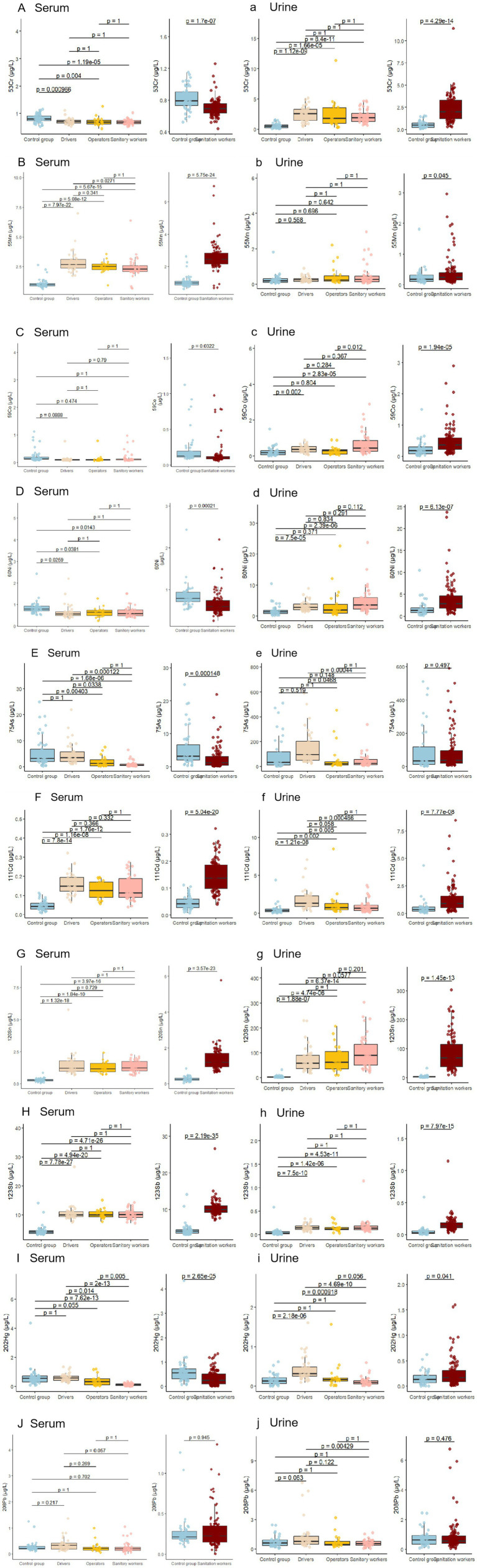
A comparative study examines the levels of 10 heavy metals in the serum and urine of sanitation workers versus a control group. Capital letters **(A–J)** represent serum concentrations of Cr, Mn, Co, Ni, Cd, Sn, Sb, Hg, and Pb for the control group, drivers, operators, and sanitation workers. Lowercase letters **(a–j)** denote the same metals in urine. The right section of the figure focuses on comparing the control group with sanitation workers, showing individual concentrations as colored dots.

Heavy metals, such as Cr, Mn, Co, Ni, As, Cd, Sn, Hg, and Pb, were detected in all human serum samples. Notably, there was a significant variation in the concentration of these different heavy metals. Except for Pb Figure 2J, the levels of nine elements exhibited significant differences between sanitation workers and the control group (Table 2). Specifically, the concentrations of Mn (2.46 μg/L), Cd (0.14 μg/L), Sn (1.19 μg/L), and Sb (9.99 μg/L) were higher in the serum of sanitation workers compared to the control group (Figure 2B,F,G,H). Conversely, the levels of Cr (0.79 μg/L), Co (0.12 μg/L), Ni (0.79 μg/L), As (3.21 μg/L), and Hg (0.55 μg/L) were elevated in the control group (*p <* 0.05). (Figure 2A,C,D,E,I)

**Table 2:** The concentration of 10 heavy metals in human serum from sanitation workers and control group (median [IQR]).

Variable (μg/L)	Overall *N* = 152	Concentration of 10 heavy metals in human serum		*p*-value
		Sanitation workers *N* = 102 (67%)	Control group *N* = 50 (33%)	
53Cr	0.71 [0.65, 0.79]	0.70 [0.64, 0.75]	0.79 [0.73, 0.91]	<0.001
55Mn	2.20 [1.07, 2.65]	2.46 [2.17, 2.84]	0.99 [0.88, 1.09]	<0.001
59Co	0.11 [0.09, 0.14]	0.10 [0.09, 0.12]	0.12 [0.11, 0.19]	<0.001
60Ni	0.66 [0.52, 0.81]	0.59 [0.49, 0.74]	0.79 [0.71, 0.93]	<0.001
75As	2.06 [0.61, 4.01]	1.18 [0.46, 3.10]	3.21 [2.02, 6.73]	<0.001
111Cd	0.10 [0.06, 0.16]	0.14 [0.10, 0.19]	0.04 [0.03, 0.06]	<0.001
120Sn	0.94 [0.33, 1.46]	1.19 [0.94, 1.70]	0.25 [0.20, 0.32]	<0.001
123Sb	9.23 [4.58, 10.60]	9.99 [9.19, 11.04]	4.07 [3.55, 4.52]	<0.001
202Hg	0.35 [0.14, 0.61]	0.25 [0.10, 0.50]	0.55 [0.31, 0.71]	<0.001
208Pb	0.22 [0.17, 0.32]	0.22 [0.15, 0.35]	0.21 [0.19, 0.28]	0.95

### 3.4 Concentration levels of heavy metals in human serum in operators, drivers and sanitary workers

In comparison to individuals engaged in other occupations, drivers exhibited elevated serum concentrations of various metals, including Mn (2.67 μg/L), Cd (0.15 μg/L), Sn (1.18 μg/L), and Hg (0.57 μg/L), as illustrated in Table 3; Figures 1B,F–H,J. Notably, Sb (10.10 μg/L) was found to be most abundant in sanitary workers. Conversely, metals such as Cr (0.79 μg/L), Co (0.12 μg/L), and Ni (0.79 μg/L) were present at higher concentrations in the serum of the control group. These differences were statistically significant. Although Pb levels were highest among drivers, this finding did not reach statistical significance (*p* > 0.05).

**Table 3:** The concentration of 10 heavy metals detected in human serum from operators, drivers, sanitary workers and control groups (median [IQR]).

		Concentration of 10 heavy metals in human serum				*p-*value
Variable (μg/L)	Overall *N* = 152	Operators *N* = 22 (14%)	Drivers *N* = 37 (24%)	Sanitary workers *N* = 43 (28%)	Control group *N* = 50 (33%)	
53Cr	0.71 [0.65, 0.79]	0.68 [0.62, 0.75]	0.70 [0.65, 0.76]	0.68 [0.63, 0.75]	0.79 [0.73, 0.91]	<0.001
55Mn	2.20 [1.07, 2.65]	2.51 [2.27, 2.73]	2.67 [2.37, 3.12]	2.29 [2.10, 2.60]	0.99 [0.88, 1.09]	<0.001
59Co	0.11 [0.09, 0.14]	0.09 [0.08, 0.11]	0.09 [0.08, 0.11]	0.11 [0.09, 0.13]	0.12 [0.11, 0.19]	<0.001
60Ni	0.66 [0.52, 0.81]	0.63 [0.50, 0.74]	0.56 [0.49, 0.67]	0.57 [0.49, 0.76]	0.79 [0.71, 0.93]	<0.001
75As	2.06 [0.61, 4.01]	1.20 [0.34, 2.60]	3.49 [2.04, 5.68]	0.52 [0.38, 0.96]	3.21 [2.02, 6.73]	<0.001
111Cd	0.10 [0.06, 0.16]	0.13 [0.09, 0.17]	0.15 [0.12, 0.20]	0.11 [0.09, 0.19]	0.04 [0.03, 0.06]	<0.001
120Sn	0.94 [0.33, 1.46]	1.12 [0.93, 1.59]	1.18 [0.96, 1.77]	1.20 [1.00, 1.71]	0.25 [0.20, 0.32]	<0.001
123Sb	9.23 [4.58, 10.60]	10.00 [9.30, 11.12]	9.97 [9.32, 11.05]	10.10 [9.03, 10.97]	4.07 [3.55, 4.52]	<0.001
202Hg	0.35 [0.14, 0.61]	0.32 [0.10, 0.50]	0.57 [0.42, 0.69]	0.10 [0.06, 0.17]	0.55 [0.31, 0.71]	<0.001
208Pb	0.22 [0.17, 0.32]	0.21 [0.15, 0.26]	0.32 [0.18, 0.41]	0.19 [0.13, 0.26]	0.21 [0.19, 0.28]	0.025

### 3.5 Concentration levels of heavy metals in human urine between sanitation workers and control group

Various heavy metals, including Cr, Mn, Co, Ni, As, Cd, Sn, Hg, and Pb, were found in all human urine samples. There was a notable variation in the levels of these metals. Six elements—Cr (1.97 μg/L), Co (0.35 μg/L), Ni (2.79 μg/L), Cd (0.92 μg/L), Sn (6.43 μg/L), and Sb (0.14 μg/L)—showed significant concentration differences between sanitation workers and the control group (*p* < 0.05), as shown in Table 4. Remarkably, these elements were more concentrated in the urine of sanitation workers than in the control group (*p* < 0.05). Although Mn, As, Hg, and Pb were also higher in sanitation workers, the difference was not statistically significant (*p* > 0.05).

**Table 4:** The concentration of 10 heavy metals in human urine from sanitation workers and control group (median [IQR]).

		Concentration of 10 heavy metals in human urine		*p*-value
Variable (μg/L)	Overall *N* = 140	Sanitation workers *N* = 102 (73%)	Control group *N* = 38 (27%)	
53Cr	1.48 [0.75, 2.69]	1.97 [1.20, 3.25]	0.56 [0.24, 0.83]	<0.001
55Mn	0.23 [0.14, 0.39]	0.24 [0.15, 0.36]	0.20 [0.11, 0.40]	0.23
59Co	0.33 [0.18, 0.54]	0.35 [0.21, 0.58]	0.19 [0.08, 0.36]	<0.001
60Ni	2.39 [1.50, 4.07]	2.79 [1.83, 4.45]	1.43 [0.79, 2.44]	<0.001
75As	37.57 [18.07, 104.66]	39.09 [19.71, 100.61]	34.29 [13.48, 110.35]	0.43
111Cd	0.66 [0.34, 1.31]	0.92 [0.53, 1.58]	0.34 [0.20, 0.59]	<0.001
120Sn	5.89 [4.03, 7.44]	6.43 [5.40, 7.67]	3.29 [2.54, 4.53]	<0.001
123Sb	0.12 [0.07, 0.16]	0.14 [0.11, 0.19]	0.04 [0.02, 0.07]	<0.001
202Hg	0.16 [0.08, 0.30]	0.18 [0.09, 0.32]	0.13 [0.07, 0.20]	0.018
208Pb	0.58 [0.41, 0.90]	0.58 [0.41, 0.89]	0.56 [0.36, 0.92]	0.43

### 3.6 Concentration levels of heavy metals in human urine in operators, drivers, and sanitary workers

In particular, operators exhibited elevated concentrations of Cr (2.37 μg/L) (see Table 5; Figure 2a), while sanitation workers showed higher levels of Co (0.41 μg/L) (Figure 2c), Ni (3.54 μg/L) (Figure 2d), and Sn (6.79 μg/L) (Figure 2g) in human urine. Drivers demonstrated the highest concentrations of As (95.29 μg/L) (Figure 2e), Cd (1.34 μg/L) (Figure 2f), Sb (0.14 μg/L) (Figure 2h), and Hg (0.31 μg/L) (Figure 2i). Additionally, Mn (0.25 μg/L) (Figure 2b) was most elevated in operators, and Pb (0.72 μg/L) (Figure 2j) was highest in drivers; however, the differences in Mn and Pb concentrations were not statistically significant (*p <* 0.05).

**Table 5:** Concentrations of 10 heavy metals detected in human urine from operators, drivers, sanitary workers and control groups (Median [IQR]).

		Concentration of 10 heavy metals in human urine				*p*-value
Variable (μg/L)	Overall *N* = 140	Operators *N* = 22 (16%)	Drivers *N* = 37 (26%)	Sanitary workers *N* = 43 (31%)	Control group *N* = 38 (27%)	
53Cr	1.48 [0.75, 2.69]	2.37 [1.35, 3.31]	1.96 [1.19, 3.33]	1.87 [1.22, 2.63]	0.56 [0.24, 0.83]	<0.001
55Mn	0.23 [0.14, 0.39]	0.25 [0.17, 0.48]	0.24 [0.16, 0.32]	0.23 [0.13, 0.36]	0.20 [0.11, 0.40]	0.54
59Co	0.33 [0.18, 0.54]	0.30 [0.14, 0.35]	0.35 [0.20, 0.53]	0.41 [0.30, 0.69]	0.19 [0.08, 0.36]	<0.001
60Ni	2.39 [1.50, 4.07]	2.18 [1.10, 3.11]	2.79 [1.85, 3.82]	3.54 [2.14, 5.30]	1.43 [0.79, 2.44]	<0.001
75As	37.57 [18.07, 104.66]	29.02 [19.00, 90.90]	95.29[41.80, 201.39]	25.36 [14.36, 46.19]	34.29 [13.48, 110.35]	<0.001
111Cd	0.66 [0.34, 1.31]	0.84 [0.66, 1.20]	1.34 [0.87, 2.35]	0.61 [0.32, 1.19]	0.34 [0.20, 0.59]	<0.001
120Sn	5.89 [4.03, 7.44]	6.37 [5.16, 7.74]	6.30 [5.78, 7.69]	6.79 [4.93, 7.56]	3.29 [2.54, 4.53]	<0.001
123Sb	0.12 [0.07, 0.16]	0.14 [0.11, 0.15]	0.14 [0.11, 0.19]	0.13 [0.10, 0.19]	0.04 [0.02, 0.07]	<0.001
202Hg	0.16 [0.08, 0.30]	0.17 [0.14, 0.32]	0.31 [0.21, 0.47]	0.10 [0.05, 0.17]	0.13 [0.07, 0.20]	<0.001
208Pb	0.58 [0.41, 0.90]	0.55 [0.39, 1.33]	0.72 [0.47, 1.09]	0.54 [0.38, 0.77]	0.56 [0.36, 0.92]	0.088

### 3.7 Concentration levels of heavy metals in human whole blood from operators, drivers and sanitary workers group

**Figure 3:**
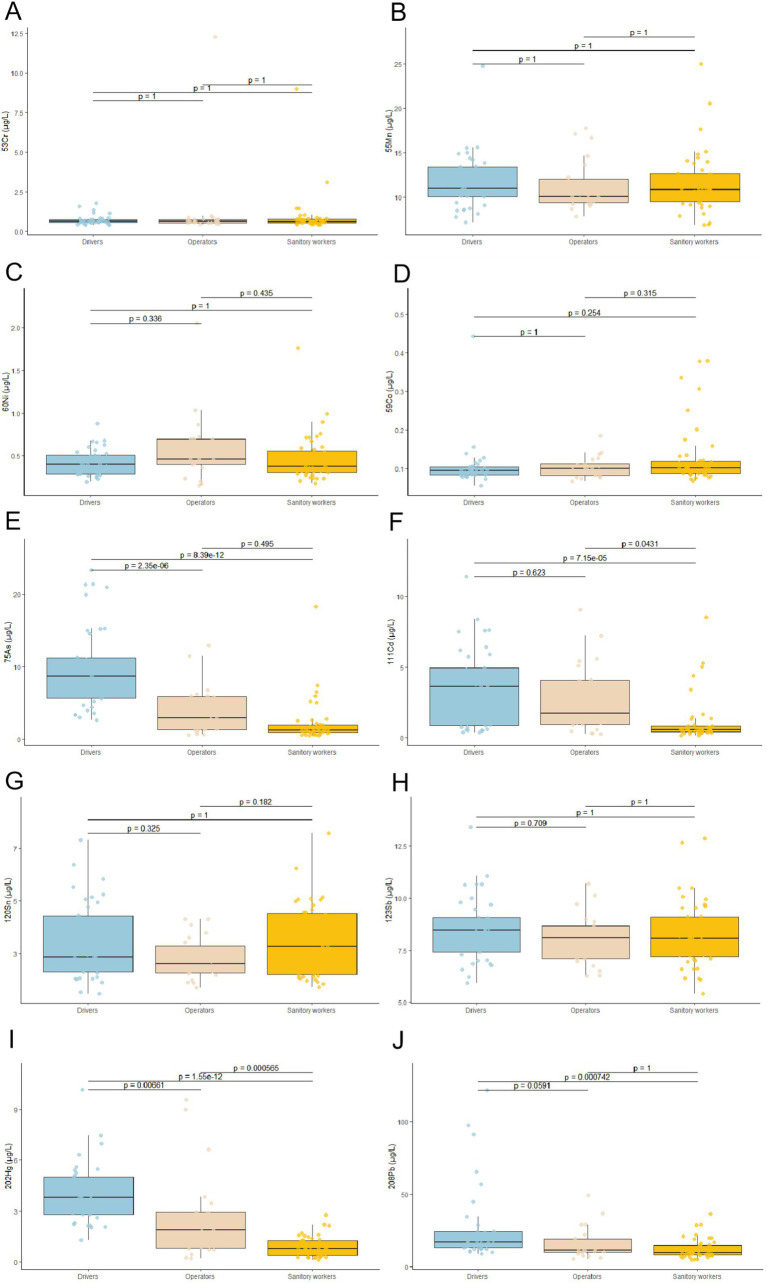
A comparative study examines the levels of 10 heavy metals in whole blood of sanitation workers versus a control group. **(A–J)** Represent whole blood concentrations of Cr, Mn, Co, Ni, Cd, Sn, Sb, Hg, and Pb for drivers, operators, and sanitation workers. The figure focuses on comparing drivers, operators and sanitary workers, showing individual concentrations as colored dots.

The concentrations of 10 heavy metals in whole blood were assessed and compared across three occupational subgroups: Operators, Drivers, and Street Sanitary Workers (see Table 6). The analysis revealed distinct exposure profiles associated with specific occupational duties. Statistically significant differences (*p* < 0.05) were identified among the groups for As, Cd, and Hg (Figures 3E,F,I). In all instances, drivers exhibited the highest concentrations, followed by operators, while street sanitary workers consistently demonstrated the lowest levels. Additionally, for Pb, the concentration in drivers (16.71 μg/L) was significantly higher than that observed in operators (11.16 μg/L). No statistically significant difference was observed between Operators and Street Sanitary Workers, with concentrations measured at 9.48 μg/L (Figure 3J). Similarly, no statistically significant differences were identified among the three groups for the other metals analyzed, including Cr, Mn, Co, Ni, Sn, and Sb (Figure 3A,B,C,D,G,H). It is important to highlight that, for all metals and across all occupational subgroups, the median concentrations recorded in this study were below the biological exposure indices (BEIs) established by the American Conference of Governmental Industrial Hygienists (ACGIH) and the limits specified by the Chinese national standard (GBZ 2.1–2019) and some recommended standards (WS/T 112–1999).

**Table 6:** Heavy metals detected in human whole blood from operators, drivers, sanitary workers [median (IQR)].

		Concentration of 10 heavy metals in human whole blood				Biological exposure indices (BEI, μg/L)
Variable (μg/L)	Overall *N* = 102	Operators *N* = 22 (16%)	Drivers *N* = 37 (26%)	Sanitary workers *N* = 43 (31%)	*p-*value	
53Cr	0.64 [0.57, 0.78]	0.66 [0.56, 0.77]	0.64 [0.59, 0.76]	0.63 [0.55, 0.80]	0.852^#^	8 (GBZGBZ 2.1–2019)
55Mn	10.85 [9.47, 13.08]	10.09 [9.42, 12.09]	10.97 [10.09, 13.38]	10.89 [9.52, 12.64]	0.691	15 (ACGIH&GBZ 2.1–2019)
59Co	0.10 [0.08, 0.11]	0.10 [0.08, 0.11]	0.09 [0.08, 0.10]	0.10 [0.09, 0.12]	0.231	5 (ACGIH)
60Ni	0.42 [0.31, 0.56]	0.46 [0.40, 0.70]	0.40 [0.29, 0.51]	0.38 [0.31, 0.56]	0.237	10 (ACGIH)
75As	3.24 [1.30, 7.67]	2.94 [1.36, 5.90]	8.70 [5.71, 11.27]	1.30 [0.95, 2.02]	<0.001	5 (HBM)
111Cd	0.96 [0.53, 3.82]	1.74 [0.94, 4.09]	3.62 [0.90, 4.96]	0.57 [0.44, 0.85]	<0.001	5 (ACGIH& WS/T 113–1999)
120Sn	2.85 [2.25, 4.10]	2.61 [2.26, 3.29]	2.86 [2.30, 4.44]	3.26 [2.21, 4.51]	0.281	–
123Sb	8.21 [7.15, 9.02]	8.09 [7.09, 8.66]	8.46 [7.41, 9.08]	8.07 [7.17, 9.11]	0.440	10 (GBZ 2.1–2019)
202Hg	1.77 [0.78, 3.40]	1.88 [0.81, 2.93]	3.82 [2.81, 5.01]	0.78 [0.40, 1.27]	<0.001	15 (ACGIH); 10 (GBZ2.1–2019)
208Pb	12.77 [9.04, 19.73]	11.16 [9.63, 18.83]	16.71 [13.03, 24.02]	9.48 [8.06, 14.75]	<0.001	400 (WS/T 112–1999)

ACGIH refers to the American Conference of Governmental Industrial Hygienists, while GBZ denotes national standards, and WS/T represents the recommended standard. HBM: Human Biomonitoring values established by the German Federal Environment Agency. HBM-I is a tolerance value below which no adverse health effects are expected. HBM-II is an action level above which there is an increased risk to health.

## 4 Discussion

The study identified significant associations between occupational factors, personal protective equipment (PPE) usage, and metal concentrations in sanitation worker’s serum, urine and whole blood. Certain factors were found to increase exposure, whereas others appeared to reduce bioaccumulation, potentially due to alterations in toxicokinetics or behavioral changes. A history of hand injuries was correlated with elevated serum, blood levels of Hg and serum levels of Pb, underscoring the critical role of skin integrity in mitigating metal absorption. Compromised skin can facilitate the entry of metals into the bloodstream, thereby increasing systemic concentrations (23, 24). Interestingly, a history of wounds on the hands and feet, as well as employment in waste management, was associated with significantly lower urinary levels of Cr, Sn, and Pb. Urinary metal concentrations are commonly used as biomarkers for recent exposure or the body’s burden of these elements. The observed inverse correlations are intriguing and may indicate altered toxicokinetics rather than decreased exposure. Chronic, low-level exposure and compromised skin integrity may modify absorption, distribution, metabolism, and excretion (ADME) processes, with metals entering through skin lesions potentially undergoing different pharmacokinetic pathways compared to those inhaled or ingested (25, 26). The use of boots, gloves and masks is correlated with elevated serum Mn levels, urinary Sb levels and whole blood Cd levels. It is plausible that workers in environments with higher dust levels are more likely to wear boots, gloves and masks consistently. Similarly, workers who utilize gloves for handling solvents may be inadvertently exposed to di (2-ethylhexyl) phthalate (DEHP), a plasticizer used in glove manufacturing, which can permeate the skin during glove use. This highlights the importance of selecting protective gloves in good quality with care, taking into account not only their chemical resistance but also the potential health risks associated with the leaching of manufacturing chemicals (27). Furthermore, longer working hours were associated with reduced serum Co levels. This inverse relationship could be attributed to varying exposure conditions or physiological factors, such as workers with extended hours performing tasks with lower Co exposure or operating in better-ventilated environments. The finding that long working hours and working at the garbage disposal site were both independently associated with elevated blood Sb levels is highly plausible. Sb is commonly used as a flame retardant in plastics and textiles, which are abundant in municipal solid waste. A longer duration in the work environment directly translates to a greater cumulative dose.

In our study, we observed elevated concentrations of Mn, Cd, Sn and Sb were higher in the serum of sanitation workers, with particularly high levels noted in the drivers’ subgroup. Sb was particularly elevated in the serum of sanitation workers. Additionally, the urine of these workers showed increased levels of Cr, Co, Ni, Cd, Sn, and Sb. Among the sanitation workers, operators exhibited a heightened concentration of Cr. While drivers demonstrated elevated levels of As, Cd, Sb, and Hg. Sanitary workers exhibited higher concentrations of Co, Ni, and Sn. The concentration levels of As, Cd, Hg in driver’s subgroup were highest, followed by operators, while sanitary workers showed lowest levels. For blood Pb, the concentration in drivers was significantly higher than that observed in operators. The concentration for all blood metals recorded in this study were below the BEI according to ACGIH, GBZ, and WS/T. Notably, the serum levels of Cr, Co, Ni, As, and Hg in the control group were higher than the case group.

Cr (8 μg/L blood, recommended by GBZ 2.1–2019) is a heavy metal with significant potential hazards to human health and the environment. It is known to enter the human body primarily through the respiratory tract, where it can undergo valence changes, leading to various toxic effects (28, 29). Once inside the body, Cr can undergo valence changes, which are critical to its toxicity. Mn (15 μg/L blood, ACGIH and GBZ) is an essential trace element involved in various physiological processes, including enzyme activation and antioxidant defense. However, excessive accumulation of Mn in the body can lead to neurological damage, a concern substantiated by several studies. Furthermore, Mn′s involvement in neurological damage is underscored by its potential to disrupt neurotransmitter synthesis and release, contributing to neurobehavioral disorders (30). Co (5 μg/L blood, ACGIH) is another trace element that, while necessary in small amounts, can be toxic at higher concentrations. Studies have shown that Co exposure can lead to adverse health outcomes, particularly affecting the cardiovascular and hematological systems (31). Ni (10 μg/L blood, ACGIH) is a prevalent allergen known to trigger allergic reactions, particularly contact dermatitis, which is a significant concern for individuals in various occupational settings, including sanitation workers. The prevalence of Ni as a common allergen is well-documented in the literature, highlighting its role in allergic contact dermatitis (32). A study found that e-waste workers’ urinary nickel concentrations (4.79 μg/L), blood Pb concentrations (101.9 μg/L) were significantly higher than those of the control group (4.02 μg/L), (44.25 μg/L) suggesting these workers may face health risks due to nickel and Pb (400 μg/L blood, WS/T 112–1999) exposure (19). Although the trend presented the same, the concentration in this study is lower (2.79 μg/L urinary Ni, 1.43 μg/L for control group; 12.77 μg/L blood Pb) compared to e-waste workers. The study conducted on teenage scavengers at a major electronic waste dumpsite in Lagos, Nigeria, provides a pertinent context for understanding the occupational exposure to heavy metals like Cd (5 μg/L, ACGIH and GBZ), which is also a concern for sanitation workers (33) which may lead to renal dysfunction and cardiovascular diseases. Furthermore, sanitation workers who hand e-waste are at risk from tin and other toxic metals like Pb, Sb (10 μg/L blood, GBZ) and Cr (34–38). Occupational exposure assessments indicate that e-waste recycling workers face insufficient protection, with high metal levels found in biological and environmental samples (38). Understanding these mechanisms highlights the risks to sanitation workers and emphasizes the need for protective measures against metal exposure. Hg (15 μg/L blood for ACGIH, 10 μg/L blood for GBZ) is a highly toxic heavy metal that damages the nervous system, kidneys, immune system, among others. In studies on workers engaged in gold extraction, mercury concentrations in their plasma, red blood cells, urine, hair, and nails were significantly higher than those in the control group (39). The occupational exposure of sanitation workers to Pb-containing waste is a significant concern due to the potential health risks associated with lead exposure. This underscores the need for stringent safety measures and monitoring to mitigate exposure risks (40).

The importance of implementing effective prevention strategies is further supported by research on occupational exposure to heavy metals in various industries (41). Similarly, research on e-waste dismantling activities emphasizes the role of behavioral science methods in reducing heavy metal exposure by improving workers’ standardized behavior habits and intentions (42). The study on iron and steel foundry workers further emphasizes the need for improved workplace ventilation and industrial hygiene practices to mitigate the accumulation of heavy metals like Pb, Cd, Cr, and Ni in workers’ blood (43). These studies collectively reinforce the argument that prevention and intervention measures are of profound importance in reducing heavy metal exposure among sanitation workers and other occupational groups. The paradoxical association between PPE use and elevated metal levels in our study is likely attributable to two key factors. Primarily, PPE serves as a proxy for high-risk tasks (confounding by indication), where workers in the most contaminated roles are mandated to use protection. Secondly, exposure could be exacerbated by inadequate PPE efficacy—including chemical permeation, material leaching, or improper removal—which may lead to inadvertent self-contamination. To mitigate exposure, a hierarchy of controls should be implemented: prioritizing engineering and administrative measures over reliance on PPE, which must be correctly selected, used, and removed through targeted training, complemented by strict post-work hygiene to prevent secondary exposure.

Based on the findings of this study, a multi-level intervention strategy is recommended to mitigate heavy metal exposure among sanitation workers. This includes implementing annual biomonitoring of Pb, Cd, Hg, and As in whole blood for high-risk roles and enhancing source segregation of hazardous waste. Engineering controls such as local exhaust ventilation and dust suppression systems should be prioritized, supplemented by administrative measures like job rotation. For personal protection, provision of task-specific PPE (nitrile gloves, N95 respirators) must be coupled with targeted training in proper use, safe doffing techniques, and workplace hygiene. Finally, strict post-shift hygiene protocols are essential to prevent take-home exposure. A comprehensive approach combining engineering controls, administrative policies, and behavior-focused protection is crucial to safeguard this essential workforce.

This study is subject to several limitations. The findings may be influenced by unmeasured confounding factors. Dietary habits which can significantly impact an individual’s health status and biomarker levels, were not accounted for. Similarly, non-occupational environmental exposures such as air pollution and key lifestyle factors (e.g., smoking status, alcohol consumption, and physical activity levels) were not assessed. Furthermore, genetic susceptibility, which can modulate metabolic pathways and individual response to exposures, was not investigated. The inability to control for or measure these variables means that their residual confounding effects cannot be ruled out, and the reported effect estimates should be interpreted with this caution in mind. The moderate sample sizes in specific subgroups (such as operators *n* = 22) may have been underpowered to detect modest effect sizes, increasing the risk of Type II error for these comparisons. A significant methodological limitation is the small size of the control group and the absence of whole blood sample from control group. To assess the absolute level and potential health significance of exposure for specific, stable metals (notably Pb), we did not rely on direct comparison with the serum control values. Instead, we contextualized our whole-blood results by comparing them to nationally and internationally established biological exposure indices (BEIs) or reference values for whole blood (ACGIH, China’s GBZ standards). Limitations also include the use of de-identified control samples precluding demographic matching and the predominantly male composition of the occupational cohort.

## 5 Conclusion

In conclusion, the findings of our study not only highlight the substantial risks encountered by sanitation workers but also emphasize the urgent necessity for comprehensive prevention and intervention strategies aimed at reducing heavy metal exposure in this occupational group. Our evidence supports the implementation of integrated measures—including environmental and biological monitoring, enhanced industrial hygiene, and behavioral interventions—which would be critical in mitigating the health risks associated with occupational heavy metal exposure. These measures not only safeguard worker health but also enhance the overall safety and sustainability of occupational environments.

## Data Availability

The original contributions presented in the study are included in the article/Supplementary material, further inquiries can be directed to the corresponding authors.

## Footnotes

1
http://www.R-project.org

## Supplementary material

Supplementary figure 1 Multivariable linear regression analysis of risk factors associated with concentration of heavy metals in human serum. # represent that the group had applied Tobit model.

Supplementary figure 2 Multivariable linear regression analysis of risk factors associated with concentration of heavy metals in human urine. # represent that the group had applied Tobit model.

Supplementary figure 3 Multivariable linear regression analysis of risk factors associated with concentration of heavy metals in human blood. # represent that the group had applied Tobit model.
