# Supplementary figures and images for: A toxic burden in Ningbo, Zhejiang Province: elevated heavy metal concentrations in sanitation workers
Source: Front Public Health. 2026 Feb 3;14:1728011. doi: 10.3389/fpubh.2026.1728011 (PMC12909531; doi:10.3389/fpubh.2026.1728011)

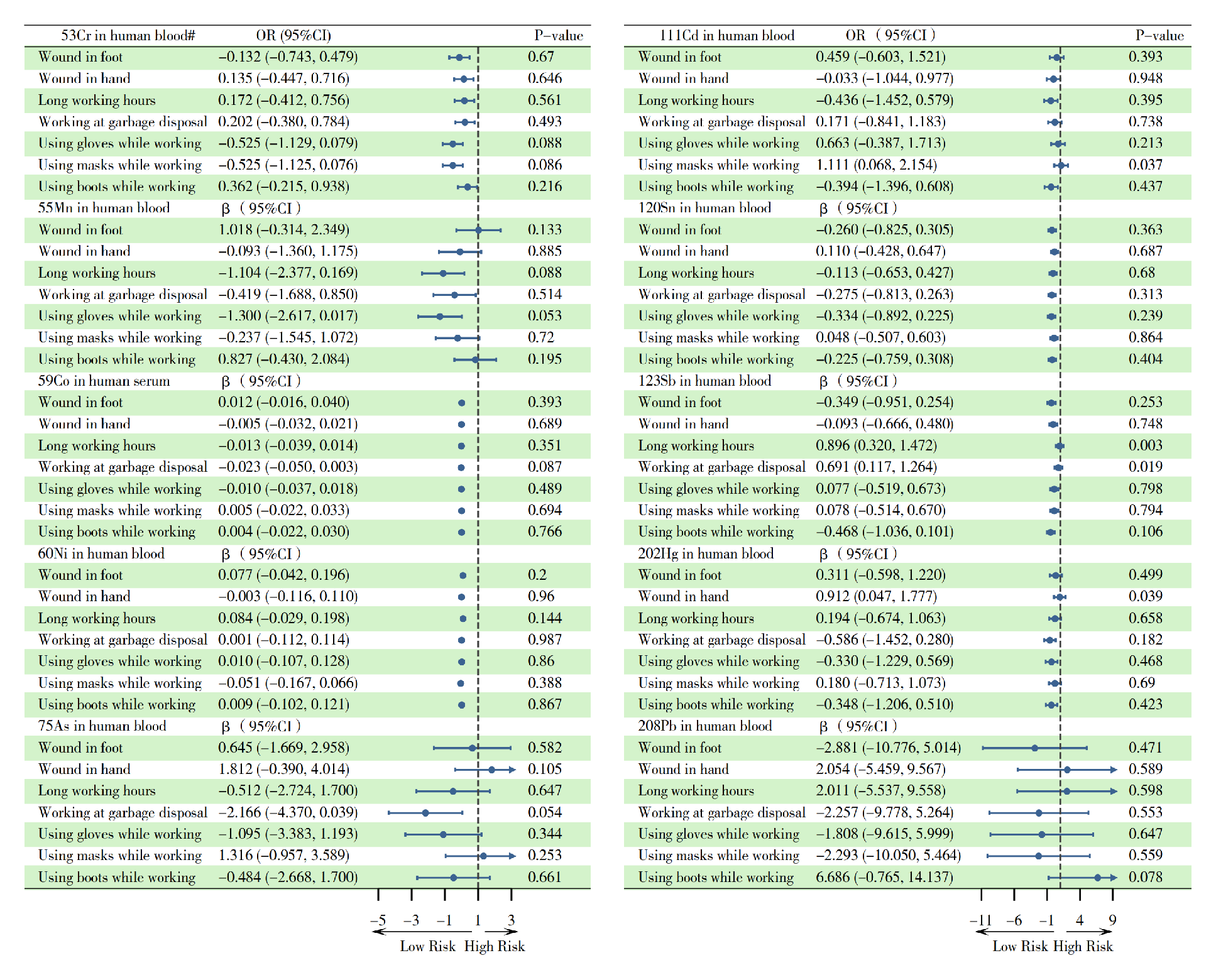

Supplement: Supplementary figure 1 — Multivariable linear regression analysis of risk factors associated with concentration of heavy metals in human serum. # represent that the group had applied Tobit model. [file Image_1.tif]

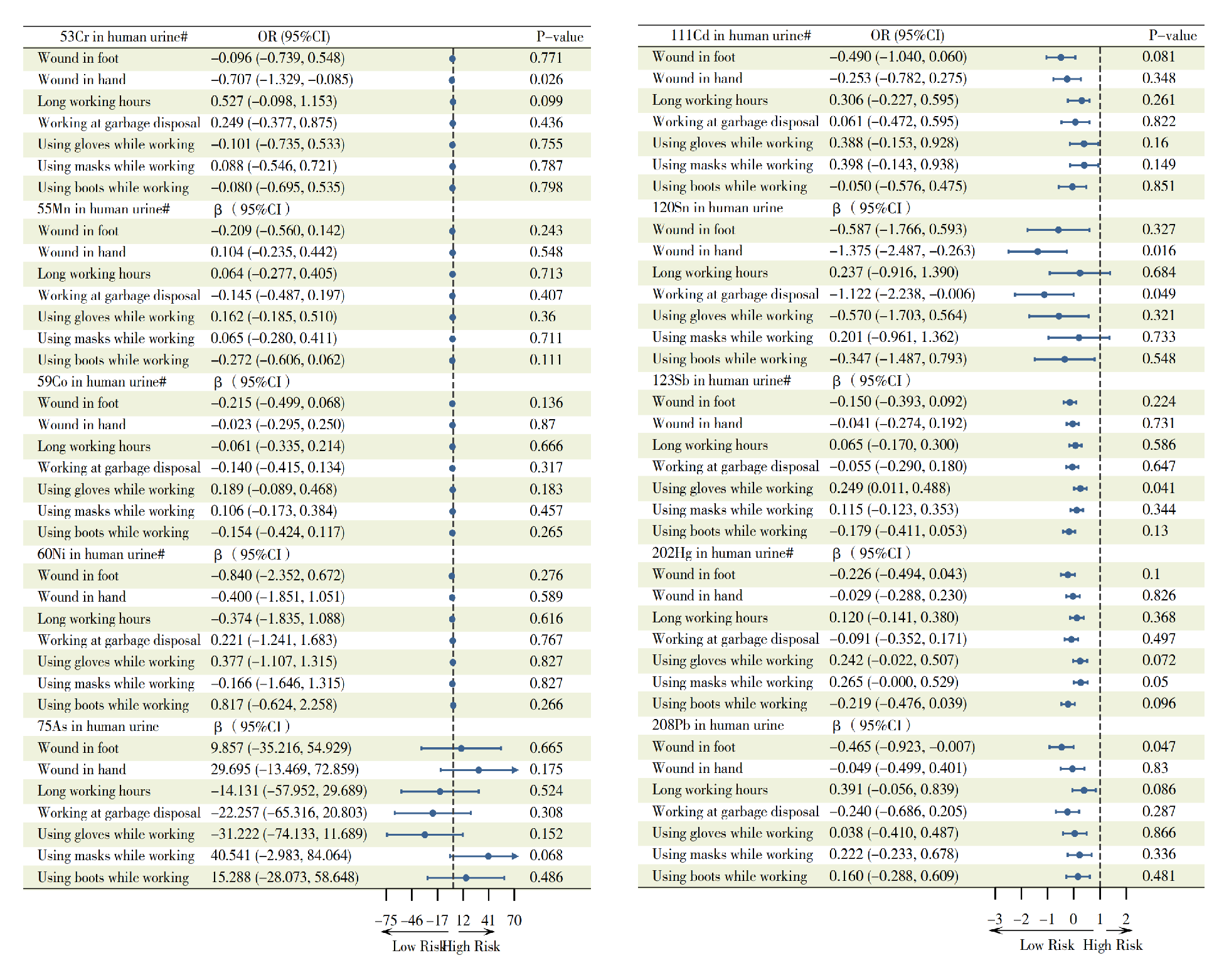

Supplement: Supplementary figure 2 — Multivariable linear regression analysis of risk factors associated with concentration of heavy metals in human urine. # represent that the group had applied Tobit model. [file Image_2.tif]

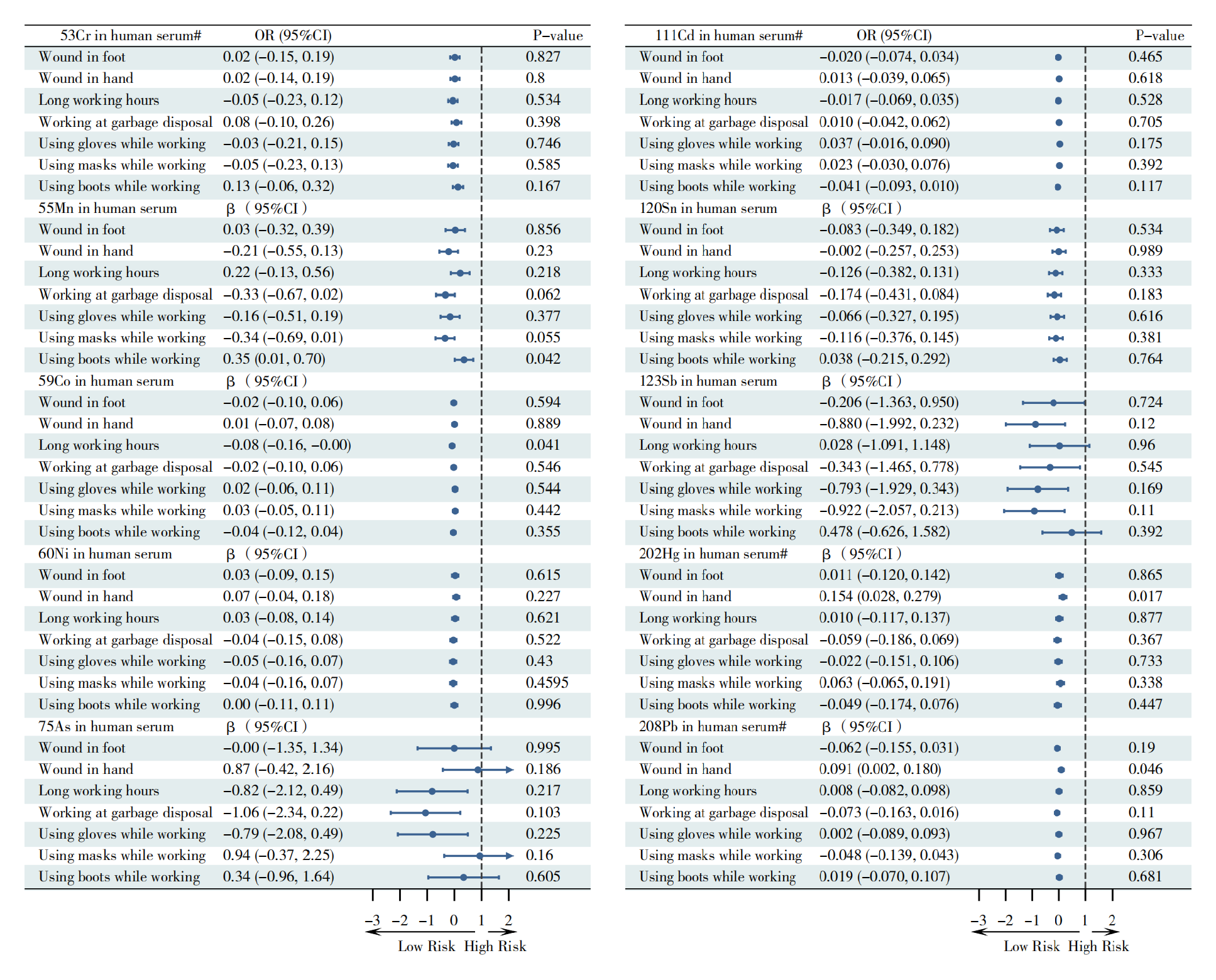

Supplement: Supplementary figure 3 — Multivariable linear regression analysis of risk factors associated with concentration of heavy metals in human blood. # represent that the group had applied Tobit model. [file Image_3.tif]
